# TRIM28 and β-Actin Identified *via* Nanobody-Based Reverse Proteomics Approach as Possible Human Glioblastoma Biomarkers

**DOI:** 10.1371/journal.pone.0113688

**Published:** 2014-11-24

**Authors:** Ivana Jovčevska, Neja Zupanec, Nina Kočevar, Daniela Cesselli, Neža Podergajs, Clara Limbaeck Stokin, Michael P. Myers, Serge Muyldermans, Gholamreza Hassanzadeh Ghassabeh, Helena Motaln, Maria Elisabetta Ruaro, Evgenia Bourkoula, Tamara Lah Turnšek, Radovan Komel

**Affiliations:** 1 Medical Centre for Molecular Biology, Institute of Biochemistry, Faculty of Medicine, University of Ljubljana, Ljubljana, Slovenia; 2 Department of Medical and Biological Sciences (DSMB), University of Udine, Udine, Italy; 3 Department of Genetic Toxicology and Cancer Biology, National Institute of Biology (NIB), Ljubljana, Slovenia; 4 Institute of Pathology, Faculty of Medicine, University of Ljubljana, Ljubljana, Slovenia; 5 International Centre for Genetic Engineering and Biotechnology (ICGEB), Trieste, Italy; 6 Cellular and Molecular Immunology, Vrije Universiteit Brussel (VUB), Brussels, Belgium; 7 Structural Biology Research Center, VIB, Brussels, Belgium; 8 Nanobody Service Facility, VIB, Brussels, Belgium; University of Michigan School of Medicine, United States of America

## Abstract

Malignant gliomas are among the rarest brain tumours, and they have the worst prognosis. Grade IV astrocytoma, known as glioblastoma multiforme (GBM), is a highly lethal disease where the standard therapies of surgery, followed by radiation and chemotherapy, cannot significantly prolong the life expectancy of the patients. Tumour recurrence shows more aggressive form compared to the primary tumour, and results in patient survival from 12 to 15 months only. Although still controversial, the cancer stem cell hypothesis postulates that cancer stem cells are responsible for early relapse of the disease after surgical intervention due to their high resistance to therapy. Alternative strategies for GBM therapy are thus urgently needed. Nanobodies are single-domain antigen-binding fragments of heavy-chain antibodies, and together with classical antibodies, they are part of the camelid immune system. Nanobodies are small and stable, and they share a high degree of sequence identity to the human heavy chain variable domain, and these characteristics offer them advantages over classical antibodies or antibody fragments. We first immunised an alpaca with a human GBM stem-like cell line prepared from primary GBM cultures. Next, a nanobody library was constructed in a phage-display vector. Using nanobody phage-display technology, we selected specific GBM stem-like cell binders through a number of affinity selections, using whole cell protein extracts and membrane protein-enriched extracts from eight different GBM patients, and membrane protein-enriched extracts from two established GBM stem-like cell lines (NCH644 and NCH421K cells). After the enrichment, periplasmic extract ELISA was used to screen for specific clones. These nanobody clones were recloned into the pHEN6 vector, expressed in *Escherichia coli* WK6, and purified using immobilised metal affinity chromatography and size-exclusion chromatography. Specific nanobody:antigen pairs were obtained and mass spectrometry analysis revealed two proteins, TRIM28 and β-actin, that were up-regulated in the GBM stem-like cells compared to the controls.

## Introduction

Several hallmarks of cancer have been described recently, which include resistance to current treatment modalities of a fraction of tumour cells that show stem-like properties, and are known as cancer stem cells [1],[2].

Gliomas, classified as astrocytomas, account for about half of all primary brain tumours [3]. Glioblastoma multiforme (GBM) are Grade IV astrocytomas that are relatively rare, but are still the most widespread and highly lethal form of glioma. These affect from 5 to 7 out of 100,000 people in the European Union [4], whereas over 10,000 new patients are diagnosed in the USA and Europe annually [5]. The American Brain Tumour Association has estimated that brain tumours are the second leading cause of cancer-related deaths in children under the age of 20 years and in males aged below 40 years [6] with the incidence of GBM increasing with age from 30 years onwards [7].

Despite all the research performed in this field, patients suffering from GBM currently have survival prognoses from 12 to 15 months [5], whereas those suffering from recurrent GBM have survival of about 6 months [8]. The standard treatment involves surgical resection, followed by chemotherapy and radiation. However, the blood–brain barrier represents a specific problem in the treatment of brain tumours, as it generally prevents the passage of molecules greater than 500 Da into the brain [9]. This thus puts serious restrictions on the use of chemotherapy, although a number of clinical trials employing more targeted treatments have been carried out with the hope of improving GBM patients' outcomes [10]–[13].

The discovery of heavy-chain-only antibodies (HCAbs) in camelids in the early 1990s [14] appears to have opened a new window of opportunity in the field of targeted treatment approaches. Due to the absence of the light polypeptide chains, HCAbs represent fully functional antigen-binding fragments that are comprised of one single domain only, known as the variable domain of the heavy chain of HCAbs (VHH) or nanobody. Nanobodies are small in size (*ca*. 2.5 nm in diameter, and *ca*. 4 nm long) [15], stable even at elevated temperatures [16] and non-physiological pH, and can be easily produced by recombinant technology. These properties make them suitable to monitor tumour biomarkers and to design improved diagnostic approaches. The high degree of sequence identity to human heavy chain variable domains (VHs) [17] suggests their lower immunogenicity when applied as therapeutics.

**Figure 1:**
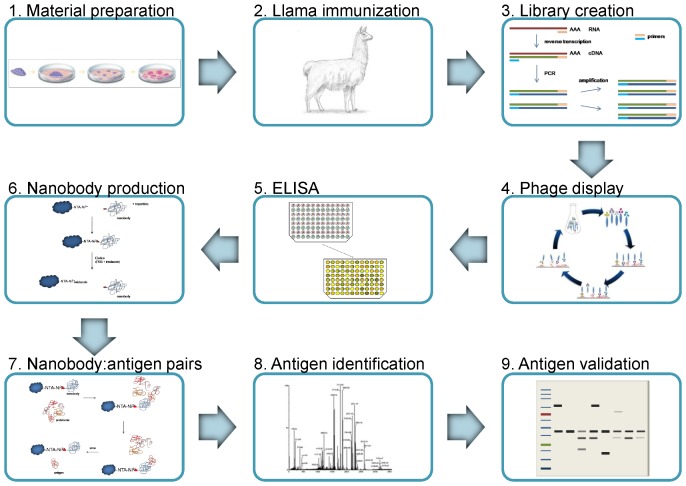
Schematic presentation of the whole workflow. 1. Material preparation – expanding a GBM cell line, laminin grown and enriched in stem-like cells, 2. Llama immunization with whole GBM cells, 3. Construction of a nanobody library, 4. Phage display cycle – enrichment of antigen specific nanobodies on various biological samples (whole protein extract from GBM tissues, membrane protein-enriched fractions from GBM tissues and GBM stem-like cell lines), 5. ELISA - selection of GBM specific nanobodies, 6. Nanobody production – large scale production and purification of specific nanobodies, 7. Nanobody:antigen pairs – immobilizing the nanobodies and binding to the corresponding antigens, 8. Antigen identification by mass spectrometry, 9. Antigen validation by Western blot. See Materials and methods for additional experimental details.

We used a phage-displayed VHH library constructed from lymphocyte mRNA from a South American camel (llama) immunized with whole human GBM cells enriched in GBM stem-like cells. The library was enriched on membrane protein-enriched fraction from two established GBM stem-like cell lines, NCH644 and NCH421K, and cell protein- and membrane protein-enriched fractions from GBM tissues of eight patients. After the enrichment, i.e. immunoaffinity selection, bacterial periplasmic extract was used in an enzyme-linked immunosorbent assay (pe-ELISA) for selecting nanobodies specific for GBM proteins. Upon incubation of these nanobodies with a protein mix from GBM stem-like cell lines, the captured cognate antigens were then identified by mass spectrometry (MS). Antigen presence in the biological samples was validated by Western blot (See Figure 1 for complete workflow).

## Materials and Methods

This study was approved by the Republic of Slovenia National Medical Ethics Committee (NMEC; http://www.kme-nmec.si/); certificate number: 92/06/12. Written informed consents were obtained from all the patients whose tumor tissues were used (a) for developing the GBM cell line (one GBM patient), and (b) for phage display and differential screening of the phage-displayed library (eight GBM patients) - provided by the University Hospital of Udine (Italy). The reference samples were obtained during routine post-mortem examinations at the Institute of Pathology of the Faculty of Medicine, University of Ljubljana. The samples were obtained from 10 individuals undergoing autopsy following appropriate legal regulations valid in the Republic of Slovenia. All the provided samples have been anonymized as required by the legislation. Also this part of the study has been granted ethical approval from the NMEC (No. 89/04/13). There are no further conditions posed by the regulation of the Republic of Slovenia regarding the anonymized use of *post-mortem* samples for research purposes.

The llama immunization and blood sampling were performed by the VIB Nanobody Service Facility (http://www.vib.be/en/research/services/nanobody-service-facility/) according to the approval of the Ethical Commission of Vrije Universiteit, Brussels, Belgium (file number 14-220-19). The animal immunization protocol is based upon on the guidelines available for Guanaco and Vicuña (llama species) as described in the Ministerial Decree of 05.03.1999 (for zoo animals) and the guidelines for farm animals used as laboratory animals described in Appendix A of the European convention for the protection of vertebrate animals used for experimental and other scientific purposes, from the European Treaty Series (ETS) 123.

### Human GBM cell-line preparation

A GBM cell line from patient tumour tissue was isolated and grown on laminin under conditions for enrichment in GBM stem-like cells following the protocols of Bourkoula *et al.* [18].

### Library construction

An adult male alpaca (23 months old) was repeatedly immunized with whole glioma cells enriched in GBM stem-like cells. Each immunization used 10^6^ whole cells, which were subcutaneously injected in the animal. In total, seven immunizations were performed, at 1 week interval. After the last immunization, 100 mL of blood was drawn from the immunized alpaca and used for the extraction of lymphocytes.

The nanobody library was constructed at the Vrije Universiteit Brussel using in-house protocols of the Nanobody Service Facility, as published by Hassanzadeh Ghassabeh *et al.* [19]. The library construction was started immediately after receiving the blood. Peripheral blood lymphocytes were isolated and their mRNA was extracted and used as template for cDNA synthesis. Two PCRs were carried out. The first PCR used the primers CALL001 (5′→3′, GTC CTG GCT GCT CTT CTA CAA GG) and CALL002 (5′→3′, GGT ACG TGC TGT TGA ACT GTT CC) to differentiate between classical Abs (*ca*. 900 bp) and HCAbs (*ca*. 600 bp). The HCAb fragments from the first PCR reaction were excised from the electrophoretic gel, purified using gel extraction kits (Qiagen), and amplified by nested PCR using the specifically designed primers A6E (5′→3′, GAT GTG CAG CTG CAG GAG TCT GGR GGA GG) and 38 (5′→3′, GGA CTA GTG CGG CCG CTG GAG ACG GTG ACC TGG GT), to amplify the variable domains of the HCAbs only (i.e., VHH or nanobodies). The VHH amplicons were purified using Qiaquick PCR purification kits (Qiagen) and ligated into a pHEN4 phagemid vector that was already digested with *Pst*I and *Not*I. This pHEN4 is a modified pHEN1 vector that was created by substituting the c-myc with an HA tag [16], and its structure is schematically represented in Hassanzadeh Ghassabeh *et al.* [19]. The nanobody library was created by transformation of the phagemids obtained into electro-competent TG1 *E. coli* cells (Lucigen).

### Sample preparation for selection

Library panning was performed on whole protein extracts from GBM tissues of eight patients (five males, three females; 40–76 years old) that underwent surgery at the University Hospital of Udine. The proteins were extracted following the procedure described by Alhamdani *et al.* [20]. Briefly, the frozen tissue (*ca*. 60 mg pooled sample) was homogenized in 300 µL extraction buffer (20 mM HEPES, pH 7.9, 10 mM glycerol, 0.5% Triton X-100, 0.5% Na-cholate, 0.5% NP-40, 0.5% CHAPS), and incubated on ice for 30 min. Upon centrifugation at 20,000×*g* for 20 min at 4°C, the supernatant was transferred into a clean microcentrifuge tube (Eppendorf, Germany) and deep frozen until used for panning.

Bio-panning was also performed on fractions enriched in membrane proteins that were extracted from the same human GBM tissue samples, and on membrane protein-enriched fractions extracted from commercially available GBM stem-like cell lines (NCH644 and NCH421K cells; Cell Line Service GmbH, Eppelheim, Germany), initially prepared and described by Campos *et al*. [21]. The NCH644 and NCH421K cells were cultivated in complete neurobasal medium: neurobasal medium (Gibco)supplemented with 20 µM/mL L-glutamine, 100 U/mL penicillin, 100 µg/mL streptomycin, 2% B-27 supplement, 20 ng/mL bFGF, 20 ng/mL EGF and 1 U/mL heparin [22].

Membrane protein-enriched fractions were obtained using two commercially available kits: ProteoExtract Transmembrane Protein Extraction kits (Novagen) and ProteoExtract Native Membrane Protein Extraction kits (Calbiochem), according to the manufacturer guidelines. The extracted fractions were analyzed by Western blot, using 1∶1500 Anti-pan Cadherin [CH-19] (abcam) and 1∶3000 monoclonal anti-GAPDH antibody produced in mouse (Sigma Aldrich) for detecting the membrane protein-enriched and cytosolic fractions, respectively, and 1∶5000 goat anti-mouse IgG (H+L) horse radish peroxidase-conjugated antibody (Jackson ImmunoResearch) as a secondary antibody. The membrane was treated with SuperSignal West Pico Chemiluminescent Substrate (ThermoScientific) and the bands were visualised using chemiluminescence and a LAS-4000 CCD camera (Fujifilm; Tokyo, Japan). The bands were analysed with the Multi Gauge version 3.2 software (for detailed Western blot procedure see Antigen validation).

The normal *post-mortem* brain samples that were used as the negative control in the enrichment of the immobilized protein extracts and for screening were provided by the Institute of Pathology, Faculty of Medicine, University of Ljubljana (Slovenia). The used brain samples originated from the hippocampus, and subventricular and periventricular areas of the brain. The membrane protein-enriched fractions from the human brains were extracted using the above-mentioned kits (from Novagen and Calbiochem). The whole protein fraction was extracted following the same protocol as for the GBM tissue samples. The concentrations of the extracted proteins from the biological samples were determined according to the Bradford method [23], using the commercial Bradford reagent (Thermo Fisher Scientific, Waltham, MA, USA).

### Phage production

An aliquot of the TG1 library that contained a representative selection of the cloned VHHs was grown in 2x Yeast extract and Tryptone (2× TY) medium containing 100 µg/mL ampicillin and 2% glucose, until the TG1 *E. coli* were growing exponentially (3–4 h at 37°C, shaking). The TG1 *E. coli* cells were then infected with an excess of M13K07 helper phages (10^12^ phages/mL) and incubated for 20 min at 37°C, without shaking. The culture was centrifuged at 1467×g for 10 min, and the supernatant was removed. The pellet was resuspended in 1 mL 2× TY medium and transferred to an Erlenmeyer flask containing 300 mL 2× TY medium with 100 µg/mL ampicillin and 70 µg/mL kanamycin. These samples were then incubated overnight at 37°C, with shaking at 225 rpm.

The phage particles were separated from the TG1 *E. coli* cells by centrifugation at 9820×g, for 30 min. The supernatant was transferred to 50 mL Falcon tubes, on ice, containing 10 mL ice-cold polyethylene glycol-6000/NaCl, and precipitated for 30 min on ice. The tubes were then centrifuged for 30 min, 2087×g, the supernatants were discarded, and the pellets (which contained the phages) were resuspended in a total volume of 1 mL phosphate-buffered saline (PBS).

### Enrichment of antigen-specific nanobodies

Library screening and enrichment of nanobodies were performed according to Hassanzadeh Ghassabeh *et al.* [19]. Both, library panning (i.e., immunoaffinity selection) and screening were performed with the same samples prepared for different protein concentrations (200 µg/mL for panning, and 2 µg/mL for screening). No more than three rounds of pannings were carried out for each biological sample.

For the bio-panning, 100 µL of GBM protein lysate (positive) and 100 µL protein lysate from normal human brains (negative) were used. After overnight coating with protein mix (200 µg/mL) and blocking of the residual protein binding sites on the plastic with 2% casein, 10^11^ of the phages produced were added to both coated wells, and incubated for 1 h at room temperature. The wells were washed several times with 0.1% PBS-Tween to remove unbound virions. The phages were eluted upon 10 min incubation with 100 mM triethylamine [24]. The 100 µL eluted phage solution was neutralized with 100 µL 1 M Tris-HCl (pH 7.4) and used in the next panning cycle.

The TG1 *E. coli* cells were grown in Luria-Bertani Miller (LB) medium for 3-4 hours at 37°C with shaking at 225 rpm. When these TG1 *E. coli* cells reach their exponential growth phase, they express F-pilus, which allows them to be infected with the eluted phage particles. Serial dilutions from the phage particles eluted from the positive (with antigen) and negative (without antigen) wells were made and used to infect the exponential TG1 *E. coli* cells. These were then incubated at 37°C for 30 min, without shaking. The TG1 *E. coli* cells were plated on both 120 mm square and 90 mm round Petri dishes, and incubated overnight at 37°C. The relative enrichment was determined by comparing the number of colonies (from the positive and negative wells) grown on the square Petri dishes, while the round Petri dishes were used for picking single colonies for subsequent ELISA screening.

The remaining phage particles that were eluted from the antigen-coated wells (positive) were mixed with exponential TG1 *E. coli* cells. After 30 min incubation without shaking, 2× TY medium containing 100 µg/mL ampicillin and 2% glucose, was added. After 20 min, 10^11^ M13K07 helper phages were also added, to infect the TG1 *E. coli* cells, and these were incubated for 20 min at 37°C, without shaking, to infect cells and to start the production of fresh phage particles. The mixture was centrifuged, the pellet was resuspended in 2× TY medium containing 100 µg/mL ampicillin and 70 µg/mL kanamycin, and the phages were produced overnight at 37°C, with shaking at 225 rpm, to be used for next round of panning. The next morning, the phages were precipitated with polyethylene glycol-6000/NaCl, as described above.

### Library screening with periplasmic-extract ELISA

Every second row of wells of the NUNC MaxiSorp plates (Thermo Scientific) was coated with GBM protein lysate (positive), with the rest of the wells coated with protein lysate from normal brain samples (negative), and these plates were incubated overnight at 4°C.

Single colonies from the round Petri dishes (see above) were picked after different rounds of panning, and these were grown in 24-well plates with 1 mL Terrific Broth (TB) and 100 µg/mL ampicillin per well, for 3–4 h. Protein expression was induced by adding isopropyl β-D-1-thiogalactopyranoside to 1 mM final concentration, followed by an overnight incubation at 37°C, with shaking at 225 rpm.

The cultures were then centrifuged at 4°C, the supernatant was removed gently and discarded, and 200 µL/well Tris/EDTA/sucrose (TES) was added. The plates were then shaken at 150 rpm for 30 min at room temperature. A minor modification was introduced using distilled (d)H_2_O instead of using a 1∶4 dilution of TES in dH_2_O (TES/4). Adding 300 µL/well dH_2_O caused an osmotic shock that released the induced proteins present in the periplasm of the TG1 *E. coli* cells. This step liberates the nanobodies, as they contain a signalling peptide that transports the polypeptide into the periplasm [25]. The plates were centrifuged for 13 min at 2087×g. Then, 100 µL/well of the supernatant was transferred to both, positive and negative wells of the coated ELISA plates. Next, addition and incubation with the primary (mouse anti-HA; Sigma-Aldrich) and secondary (goat anti mouse IgG, whole molecule; Sigma-Aldrich) antibodies followed. After this, alkaline phosphatase substrate (Sigma-Aldrich) was added, which allowed visualisation of the specifically bound proteins. The absorbance was measured at 405 nm.

A clone was considered positive if its periplasmic extract yielded an ELISA signal at least twice of that without antigen coating. The TG1 *E. coli* colonies of the nanobodies that had good ELISA signals were amplified using colony PCR, and sequenced at the Genetic Service Facility (VIB, Antwerp).

### Nanobody recloning

Three nanobodies were chosen for large-scale production, and these were recloned into the pHEN6 expression vector to extend the nanobody with a His tag [26], [27].

Single colonies of the clones were amplified by PCR using the A6E and 38 primers (framework1 and framework4 primers, respectively). The PCR products were purified using PCR purification kits, and digested overnight at 37°C with *Pst*I and *Eco91*I (both from Thermo Scientific). The products were ligated for 2 h at room temperature into pHEN6 vector. Electro-competent *E. coli* WK6 cells were transformed with the ligation reaction. The selection was made on LB agar plates containing 100 µg/mL ampicillin and 1% glucose. Five randomly chosen clones from each nanobody were amplified by colony PCR, and sequenced at the Genetic Service Facility (VIB, Antwerp). Individual bacterial clones that had exactly the same sequence as the original nanobody clones were chosen for the large-scale expression.

### Nanobody expression and purification

The expression and purification procedures were carried out as described by Vincke *et al.* [28] and Chames [29]. The procedure described is applicable for the expression of a single nanobody.

After recloning, five single colonies for each nanobody clone were picked and inoculated into 15 mL LB medium with 100 µg/mL ampicillin. These were grown overnight at 37°C, with shaking at 225 rpm. These five overnight cultures were pooled in one tube, and 5 mL of this culture was equally distributed into five shaker flasks, each containing 330 mL TB with 100 µg/mL ampicillin, 0.1% glucose and 2 mM MgCl_2_. The flasks were shaken at 200 rpm for 3–4 h at 37°C. Protein expression was induced by adding isopropyl β-D-1-thiogalactopyranoside to a final concentration of 1 mM, and the flasks were shaken overnight at 28°C at 200 rpm.

Next day, the culture was centrifuged for 8 min, at 14°C, 9820×g. The supernatant was discarded, and the pellets of all five cultures pooled. They were resuspended in 20 mL TES until the suspension became homogeneous, which was then shaken for 1 h in a cold-room at 4°C. Osmotic shock was then caused by adding 40 mL TES/4 to this suspension, which was shaken for another 2 h at 200 rpm in the cold-room at 4°C. Five hundred microliters of 2 M MgCl_2_ was added to the centrifuge tube, and the suspension was mixed and centrifuged for 30 min at 9820×g. The supernatant was transferred to two 50 mL tubes (Falcon) that already contained approximately 5 mL Ni^+^-NTA agarose (Ni-beads, Qiagen)/tube. The Ni-beads were previously washed three times with 20 mL PBS. The periplasmic extract was incubated with these Ni-beads overnight in a cold-room at 4°C, with shaking at 200 rpm.

A second overnight periplasmic extraction is optional, to obtain a higher protein yield. Here the samples from both extractions are pooled and equally distributed into four 50 mL tubes (Falcon) that already contained Ni-beads, for the incubation for 1 h in a cold-room at 4°C, shaking at 200 rpm. These were then centrifuged for 7 min at 394×g. The supernatant was discarded, and 45 mL/Falcon PBS was added to the beads, which were then shaken for 1 h in the cold-room at 4°C. The tubes were centrifuged and the supernatant was removed, leaving approximately 5 mL liquid on the resin.

The expressed nanobodies were purified using immobilised metal-affinity chromatography (IMAC). A filter was put in an empty PD-10 column, and the periplasmic extract with the Ni-beads was poured into the column. The column was washed with 20 mL PBS and drained. 10 mL freshly prepared PBS with 0.5 M imidazole was used to elute the expressed nanobodies, with this elution repeated three times. The protein concentration of the combined eluates was measured using a NanoDrop spectrophotometer. The proteins were then concentrated using Vivaspin concentrators to obtain a final nanobody concentration of 1 mg/mL. The expressed proteins were separated by SDS-PAGE and visualized after staining with Coomassie blue R-250.

These IMAC-purified nanobodies were additionally cleaned using size-exclusion chromatography, with an ÄKTA purifier (GE Healthcare) and the Unicorn software. The samples were run on a Sephadex S75 column using filtered PBS (pH 8.0) as the mobile phase, at a flow-rate of 2 mL/min. The eluate was collected in 1.5 mL microcentrifuge tubes (Eppendorf), and those corresponding to the nanobodies were collected in separate 50 mL tubes (Falcon) for each nanobody. The protein concentration was measured on a Synergy H4 Hybrid Reader. Using dilution in PBS, the nanobodies were brought to a final concentration of 1 mg/mL (when possible), and were stored at 4°C.

### Obtaining nanobody:antigen pairs

Sample preparation and MS analysis was performed according to the Generic IP protocol established by the Proteomic Facility at the International Centre for Genetic Engineering and Biotechnology (ICGEB) in Trieste, Italy (https://sites.google.com/site/icgebproteomics/documents).

Two of the nanobodies were used at 1 mg/mL, and one at 0.2 mg/mL. The concentration of the extracted membrane proteins from the GBM stem-like cell lines was 3 mg/mL.

Ni-beads (400 µL) were washed three times with 1 mL PBS. After the last wash, the Ni-beads were resuspended in 2 mL PBS and equally distributed into six microcentrifuge tubes (Eppendorf). The nanobodies were incubated with the Ni-beads for 1 h at 4°C on a rotating platform (positive samples). Three of the microcentrifuge tubes were left without nanobodies, considered as ‘negative’ samples, and these were treated identically to the Ni-beads with the nanobodies. The Ni-beads with the nanobodies were briefly centrifuged, the supernatant was discarded, and the extracted membrane proteins were added. Here, 500 µL/tube protein lysate was added to one ‘positive’ and one ‘negative’ microcentrifuge tube of the same nanobody. These were incubated again at 4°C for 1 h, while rotating. After incubation, both suspensions were centrifuged, the supernatants (protein lysate) were placed into new microcentrifuge tubes (for the second nanobody) and incubated for 1 h in the cold-room at 4°C, while rotating. The pellets (the nanobody:antigen pair) from the positive and negative microcentrifuge tubes of the first nanobody were washed three times with 1 mL 0.1% PBS-Tween, transferred to new tubes, and washed three times again with 1 mL PBS. The microcentrifuge tubes were briefly centrifuged, the supernatants were removed, and the pellets were kept on ice. The same procedure was repeated with the second and third nanobody.

### Sample preparation for mass spectrometry analysis

All of the pellet samples from the nanobody:antigen pairs were digested with trypsin solution (2 µL porcine trypsin (Promega) in 100 µL 20 mM triethylammonium hydrogen carbonate buffer), overnight at room temperature.

After the overnight digestion, the samples were centrifuged and the supernatants were transferred to a clean microcentrifuge tube. Then 25 µL 20 mM triethylammonium hydrogen carbonate buffer was added to the Ni-beads, which were centrifuged, and these supernatants were combined with the first supernatants. Then, 1 µL Tris(2-carboxyethyl)phosphine (as a reducing agent; Thermo Scientific) was added to each of the microcentrifuge tubes, which were incubated for 5 min at room temperature. Then, 1 µL chloroacetamide was added to each sample, and these were incubated for 1 h at room temperature. In order to elute any tryptic fragments that remained bound to the nanobodies, the pelleted Ni-beads were washed once with 1 mL PBS/tube, centrifuged, and the supernatants were removed. Then 25 µL 6 M urea was added to each microcentrifuge tube. The samples were centrifuged briefly, and the supernatants containing the eluted antigens were transferred to clean microcentrifuge tubes. The same volume of urea was added two more times to each microcentrifuge tube, and after brief centrifugation, the supernatants that were treated with the same nanobody were pooled. All of the supernatants were purified using Empore C18 filters. These filters were inserted into pipette tips, wet with acetonitrile, and equilibrated with 0.1% trifluoroacetic acid in water. The samples were loaded onto the filters, washed with 0.1% trifluoroacetic acid and eluted into new microcentrifuge tubes using 65% acetonitrile with 0.1% trifluoroacetic acid. The samples were finally dried for 5 min at 95°C. Prior to the analysis with MS, each sample was resuspended in 25 µL 0.2% trifluoroacetic acid.

### Mass spectrometry analysis and antigen identification

The MS analysis and antigen identification were performed at the Proteomic Facility at the ICGEB in Trieste (Italy). The purified samples were analysed by liquid chromatography–tandem MS (LC-MS/MS), using an Easy-nLC system connected to an Amazon electron-transfer dissociation ion trap (both instruments from Bruker). The LC was developed using a 75 min gradient from 0% to 80% methanol in 0.1% formic acid. The resulting MS/MS spectra were searched against a human database using the X!tandem and MASCOT search engines, allowing for a 5% false-discovery rate. This led to the identification of two antigens.

### Antigen validation

The antigens captured by the nanobodies for the MS identification were validated by Western blotting (WB). Approximately 10 µg protein extracts from the biological samples (from the GBM tissues, the GBM stem-like cell lines, and the *post-mortem* human brain samples) were separated by 12% SDS PAGE. An Immobilion-P Transfer Membrane (Millipore) was activated by incubating it for 15 s in methanol, followed by 2 min incubation in dH_2_O, and then placing it in WB transfer buffer (25 mM Tris, 192 mM glycine, 10% methanol) until use. The WB transfer was approximately 1 h at 100 V.

The proteins on the WB membrane were stained using Ponceau S and blocked in 5% PBS-milk for 1 h at room temperature, with shaking. To detect TRIM28 in the selected biological samples, the membrane was incubated with the SAB1404789 monoclonal anti-tripartite motif containing protein (TRIM28) antibody (1∶1500; Sigma Aldrich) at 4°C, overnight with shaking. After overnight incubation, the Western blot membrane was incubated with the A5441 monoclonal anti-β-actin antibody (1∶1500; Sigma Aldrich) for 1 h at room temperature, with shaking. In order to have an internal control, the Western blot membrane was incubated with the monoclonal anti-GAPDH antibody produced in mouse (1∶3000; Sigma Aldrich) for 1 h at room temperature, with shaking. The incubation with the secondary antibody, a goat anti-mouse IgG (H+L) horse radish peroxidase-conjugated antibody (1∶5000; Jackson ImmunoResearch), was also for 1 h at room temperature. SuperSignal West Pico Chemiluminescent Substrate (ThermoScientific) was spread over the membrane after the last incubation, and the bands were visualised using chemiluminescence and a LAS-4000 CCD camera (Fujifilm; Tokyo, Japan). The bands were analysed with the Multi Gauge version 3.2 software. The band intensities of the antigens, β-actin and TRIM28, were normalized to the band intensities of the internal control, GAPDH. The numerical value of the relative band intensity of the antigens was calculated as the ratio between the arbitrary units (AU) of the antigens and the arbitrary units of the internal control.

## Results and Discussion

The similarities and differences in the sequences and structures of VHH and human VH, and the specific advantages of nanobodies over classical antibodies for particular applications have been described [30], [31]. Nanobodies have a low molecular weight (14 kDa) [32], and they are small, very stable (they can be stored for months at 4°C, and even longer at −20°C), and are resistant to thermal and chemical denaturation [33]. Their size and shape allow them to recognize sites that are hidden or cryptic for interactions with classical antibodies. Above all, the immunization step of camelids and the cloning of the repertoire of the nanobodies, each nanobody as a single exon of about 350 bp gives access to *in vivo,* affinity matured target specific binders. In contrast, immunization of all other species necessitates the separate cloning of the VH and VL repertoires that in a subsequent step are joined randomly in a scFv of Fab construct whereby the original affinity matured VH-VL pairs become most likely scrambled. Hence, it is more difficult to retrieve stable high affinity binders from such libraries. In addition, the sequence similarity with human VH and their rapid blood clearance are predictive of low immunogenicity. Indeed, immune responses have never been detected in mice injected with nanobody constructs [34]. The major reason why they were chosen for possible therapeutic approaches for brain tumours is the presence of the blood–brain barrier, which limits the traffic to and from the brain. Nanobodies have been reported able to cross the blood-brain barrier and penetrate into brain cells [35]. Although the capillaries of the glial tumours are more leaky than normal brain capillaries [36], only a small percentage of drugs can pass through the blood–brain barrier at pharmacologically significant levels [37]. Up to now, there are a number of diseases such as depression, epilepsy and chronic pain, for which therapies with small molecules proved efficient, but unfortunately in this respect cancer has not been studied to any great extent [38].

We aimed to identify nanobodies directed against novel biomarkers, specific for GBM stem-like cells that would prove valuable in diagnostics and, in the future, as therapeutics. Alpaca immunization was performed with whole glioma cells to elicit preferentially an immune response against the cell-surface proteins. During the nanobody library construction, the agarose gel after the first PCR of the animal lymphocyte cDNA showed two separate bands that corresponded to the two antibody types in camelids, the upper band of around 900 bp indicating the presence of heavy chain of classical antibodies, whereas the lower band, around 600 bp, was indicative of the heavy chain of HCAbs. The noted 300-bp size difference corresponds to the presence of the CH1 domain in the classical antibodies, which is absent in the HCAbs [31]. After a nested PCR using the 600 bp DNA fragments as template to amplify the VHHs only, ligation to phage display vectors, transformation into electro-competent cells and plating on selective petri dishes, yielded a nanobody library of some 10^8^ individual transformants, which is consistent with the average size of recombinant VHH immune libraries [39]. The insert in the pHEN4 plasmid from 30 randomly chosen *E. coli* clones of this library was amplified by PCR, which showed that 80% of the clones had an insert in their pHEN4 vector with the size of a nanobody gene.

Bio-panning (i.e., immunoaffinity selection) is most successful when using a purified antigen immobilized on immunotubes or wells of microtitre plates. When using a complex sample like a protein extract from a tissue, the selection can be particularly difficult due to a limited amount of specific antigens in the sample. In addition, an enrichment of antibodies with specificity for non-relevant antigens may also occur. However, our selection was performed on a very heterogeneous panel of antigens (total protein extract and membrane protein-enriched extract from GBM tissues, and membrane protein-enriched extract from GBM stem-like cells) as we aimed to target a broader spectrum of novel antigens.

**Figure 2:**
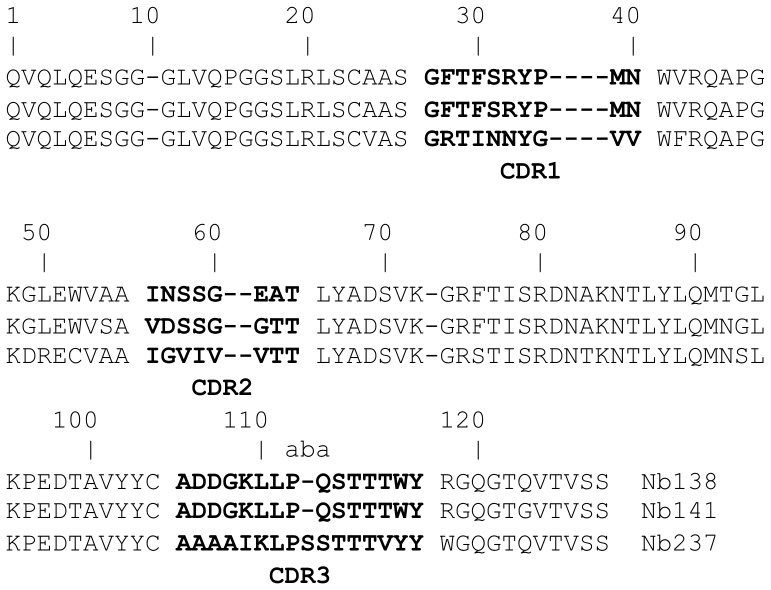
Sequences of the nanobodies that were chosen for the large-scale production. The amino acid sequence of the CDR3 regions of the nanobodies is given in alphabetic order.

A large number of colonies from different rounds of panning were screened for all of the samples. The best results were obtained from the panning and screening of the membrane protein-enriched fractions from the GBM stem-like cell lines. It is possible that these cell lines provide a more homogeneous biological sample, whereas besides tumour-cell proteins, the tissue extracts may have also contained blood vessels and other constituents of the tumour stroma, present also in the healthy tissue. Periplasmic extract ELISA (i.e., an ELISA with periplasmic proteins from bacteria) was the preferred analysis method, because the nanobodies cloned in pHEN4 have a PelB signalling peptide at the N-terminus that transports these proteins to the bacterial periplasmic region [25], [40]. In this way, most of the cytosolic proteins were avoided and the chance for false-positive signals was reduced. The HA-tag at the C-terminus of the periplasmic-expressed nanobody was used for the ELISA detection. From a number of nanobodies that displayed good ELISA signals, only three (named as Nb138, Nb141, Nb237) had amino-acid sequences (Figure 2), which clearly showed that all three domains are derived from a HCAb. The framework 2 region of Nb237 has the imprint of a VHH with F42 and R50. The C52 is surprising, although we expect that it will not lead to disulphide bond formation, as the amino acid in this position is normally covered by the long CDR3 loop. The framework 2 regions of the two other clones share a VH imprint with V42, L50 and W52. However, the R118 clearly indicates that also this domain cannot associate with a VL domain, and therefore they should originate from a HCAb as well. The CDR3 sequences of these two molecules are identical, and this predicts that they may target the same epitope [41]. The amino-acid differences between these clones in their CDR2 regions indicate that their affinities might be different, or that their binding to the target might be affected differentially by post-translational modifications of the antigen.

These three nanobodies (Nb138, Nb141, Nb237) were chosen for large-scale production. The recloning into pHEN6 was necessary to introduce the His tag, which allows for their purification through IMAC (see Materials and methods) prior to the use of a size-exclusion chromatography step. This way purified nanobody fractions were pooled and applied to 12% SDS-PAGE. Staining with Coomassie blue revealed the presence of a single protein band at the expected molecular weight (14 kDa) for a nanobody.

These nanobodies were then used to capture antigens from a heterogeneous protein extract, which were then sent for MS analysis. This MS analysis identified two antigens, TRIM28 and β-actin, specific for Nb237 and Nb141, respectively. The third nanobody (Nb138) yielded inconclusive spectra. As stated above, Nb138 and Nb141 have the same CDR3 sequence, which suggests they would bind to the same antigen [41]. As the target antigen for Nb138 could not be identified with MS, this means that the differences in the CDR2 regions between these two nanobodies (Nb138 and Nb141) might lead to a too low antigen capturing capacity for Nb138. Both of the antigens identified, TRIM28 and β-actin, belong to the group of internal cellular proteins and are discussed separately below. Identifying internal cellular proteins after selecting on membrane protein-enriched fractions may also be a result of an incomplete separation between the membrane protein-enriched and cytosolic fractions from the samples. Western blot analysis of the extracted protein samples showed the presence of cytosolic fractions in all of the samples. The signal from the membrane protein-enriched fractions was significantly stronger in the corresponding protein samples when compared to the cytosolic fractions originating from the same biological samples (see Figure S1).

Alpaca immunization was carried out with whole glioblastoma cells enriched in GBM stem-like cells, to raise antibodies against cell-surface markers. Yet, our data show that from the immune library, after phage display selections on GBM lysates, we were only able to retrieve nanobodies that recognise intracellular proteins. This might be due to the lower stability of the membrane proteins in the blood and/or the lysis of the glioma cells inside the alpaca during the course of the immunization. Hence, in the immune response, the less abundant, differentially expressed cell-surface antigens are overpowered by the overexpressed internal cellular proteins, as well as in the panning and screening procedures.

**Figure 3:**
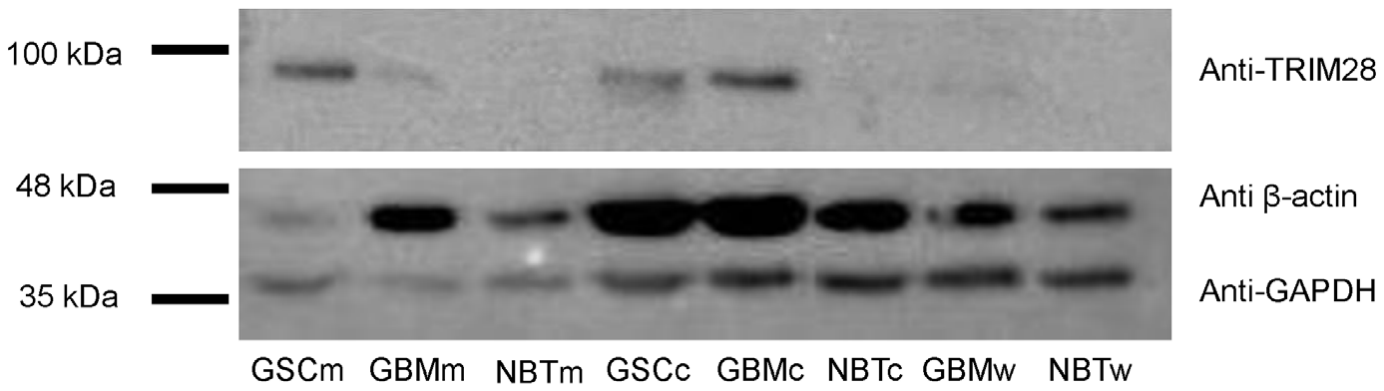
Antigen validation. Western blot membrane after probing with a monoclonal anti-GAPDH antibody (36 kDa band) used as an internal control for amount of protein loaded in each lane, a monoclonal anti-β-actin antibody (48 kDa band) and monoclonal anti-TRIM28 antibody (100 kDa band). Samples: GSCm – membrane protein-enriched extract isolated from GBM stem-like cell lines; GBMm – membrane protein-enriched extract isolated from GBM tissues; NBTm – membrane protein-enriched extract isolated from human brain samples; GSCc – cytosolic/nuclear protein fraction isolated from GBM stem-like cell lines; GBMc – cytosolic/nuclear protein fraction isolated from GBM tissues; NBTc – cytosolic/nuclear protein fraction isolated from human brain samples; GBMw – whole protein extract from GBM tissues; NBTw – whole protein extract from human brain samples.

**Figure 4:**
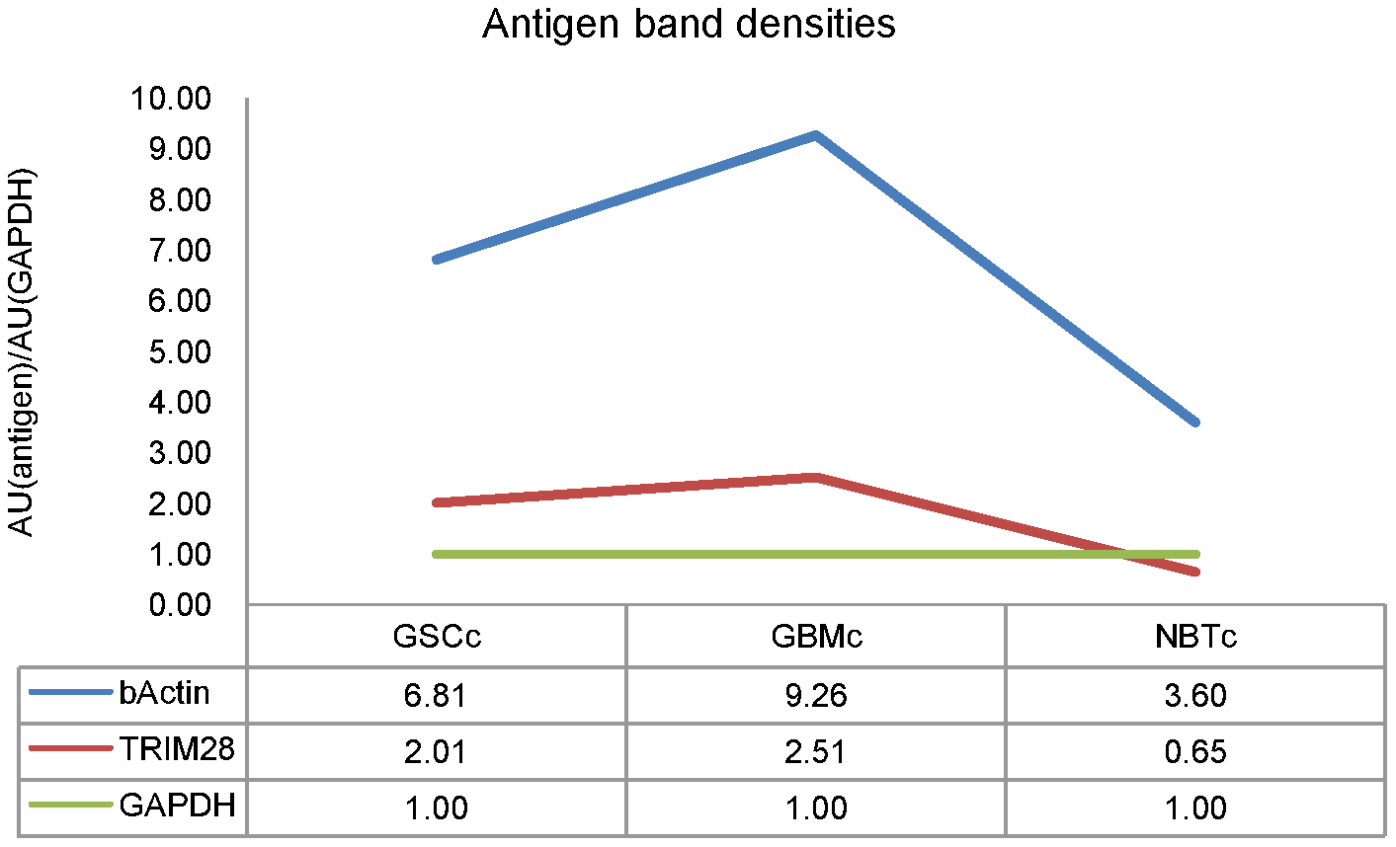
Relative band intensities of antigens validated with Western blot. The relative band intentisy of the antigens, TRIM28 and β-actin, was calculated as the ratio between arbitrary units of the band of the antigen and the arbitrary units of the band of the internal control, GAPDH [AU(antigen)/AU(GAPDH)]. Samples: GSCc – cytosolic/nuclear protein fraction isolated from GBM stem-like cell lines; GBMc – cytosolic/nuclear protein fraction isolated from GBM tissues; NBTc – cytosolic/nuclear protein fraction isolated from human brain samples.

The WB validation of the selected antigens (Figure 3) revealed TRIM28, at approximately 100 kDa, in the extracted proteins from GBM stem-like cells (in both membrane protein-enriched and cytosolic/nuclear fractions) and from GBM tissue samples (in cytosolic/nuclear fraction). The calculated relative band intensity showed a threefold increase of the TRIM28 level in the cytosolic fraction from GBM stem-like cell lines – GSCc (relative band intentisy, 2.01) and GBM tissues – GBMc (relative band intensity, 2.51) when compared to the cytosolic protein fraction from the normal brain tissue samples – NBTc (relative band intensity, 0.65), data shown in Figure 4.

β-actin is a highly abundant protein with a molecular weight of 42 kDa. The Western blot showed its presence in all of the samples, as it can be seen on Figure 3. However, the band intensities demonstrated its unequal distribution throughout the samples. The calculated relative band intensity showed a two-fold increase of the β-actin levels in the cytosolic fraction from the GBM stem-like cell lines – GSCc (relative band intensity, 6.81) compared to the cytosolic fraction from the normal brain tissue samples – NBTc (relative band intensity, 3.60). In addition, the levels of β-actin showed a threefold increase in the cytosolic fraction originating from GBM tissues – GBMc, compared to the β-actin levels in the cytosolic fraction of the post mortem brain tissue samples – NBTc, relative band intensities 9.26 and 3.60, respectively (Figure 4).

### TRIM28 as a tumour marker

**Figure 5:**
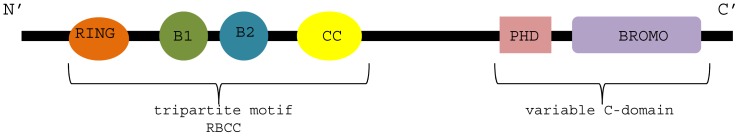
Schematic presentation of the structure of TRIM28. The conserved RBCC motif is present at the N-terminal domain; the variable C-domain includes the PHD and BROMO domains. Image adapted from Hatakeyama *et al*. [45]. Author's approval was obtained for the use of this image; original image is given in Figure S2.

TRIM28 – also known as TIF1β (transcriptional intermediary factor 1-β) and KAP1 (KRAB-interacting protein 1), is a protein that belongs to the tripartite motif (TRIM) family (also known as the RBCC family) that at the N-terminal region is composed of three conserved zinc-binding domains, a RING finger (R), B-boxes (B-box Type 1 (B1) and B-box Type 2 (B2)), and a coiled-coil region (CC). To date, about 70 members of the TRIM family are known. Their structures were explained in detail by Micale *et al.* [42]. TRIM proteins vary in the number of B-boxes and the C-terminal domain. Indeed, the TRIM family can be subdivided into 11 different groups based on the composition of the highly variable C-terminal region. The 6^th^ subfamily of the TRIM proteins, due to the presence of C-terminal BROMO and PHD domains (Figure 5), consists of three TIF1 members, TIF1α, TIF1β, TIF1γ, which are also known as TRIM24, TRIM28 and TRIM33, respectively, and found to display high sequence homology. Their well-conserved PHD and BROMO domains were found widely distributed among nuclear proteins acting at the chromatin level and involved in the control of gene expression through regulation of the transcriptional activity of numerous sequence-specific transcription factors [43], [44]. Alteration of these proteins can affect transcriptional regulation, cell proliferation and apoptosis, and members of the 6^th^ TRIM subfamily, otherwise involved in an assortment of cellular processes such as cell growth, development and differentiation [45] are also thought to be important regulators of carcinogenesis. Namely, TRIM24 and TRIM33 have been described to acquire oncogenic activity upon chromosomal translocations when the RBCC motif is fused to another protein [46], [47], whereas TRIM28 is believed to be mediator of p53 suppression by promoting p53 ubiquitylation and degradation [48].

In 2012, Chen *et al.* [49] hypothesised that in the early stages of lung adenocarcinomas, the levels of TRIM28 are up-regulated for protective reasons; i.e., to control cell growth and suppress tumour development. More recently, Liu *et al.* [50] reported that TRIM28 is a possible marker for the detection of circulating cancer cells, and provided evidence for a role for TRIM28 in metastasis development and prognosis in early stage non–small-cell lung cancer (NSCLC). In this study, 35 of 48 patients with NSCLC had overexpressed *TRIM28* mRNA levels, while the rest of these showed underexpressed *TRIM28* mRNA levels, compared to non-cancerous tissue. Liu *et al.* [50] thus proposed that TRIM28 might be involved in tumour-cell migration, and tumour invasion and progression. Even though the findings of Liu *et al.* [50] and Chen *et al.* [49] are contradictory, as lung cancers are the cause for the occurrence of 65% secondary GBMs [51], the correlation of TRIM28 to GBM shown in the present study is an interesting discovery that deserves further being investigated.

### β-actin as a tumour marker

β-actin is a highly abundant cytoskeletal protein spread throughout the peripheral cell regions and the leading edges of motile cells. It is involved in cell migration and division, wound healing, embryonic development, and immune responses [52]. As a result of its constant expression levels, β-actin has been used as an internal control for normalisation in gene expression studies [53]. However, more recently, its use as an internal control has been questioned due to the growing evidence that the expression levels of β-actin vary under different conditions. β-actin has been shown to be up-regulated in liver, gastric, colorectal, lung cancers and in melanomas [54]. The overexpression of β-actin in cancers suggests that it may have altered function in carcinogenesis [55], including of glioblastoma as lung cancers and melanomas are primary tumours that are responsible for the occurrence of secondary GBMs.

Cell migration is important during the whole of the life span of vertebrates, and especially during embryonic development. This is a process that requires changes in the actin cytoskeleton, and β-actin polymerisation and formation of invading structures. Recent studies have shown that β-actin polymerisation can promote cell motility, invasiveness and metastasis. Le *et al.* [56] showed that β-actin is localised in the pseudopodia of invasive cells, but absent in non-invasive cells. These data are further supported by the studies of Nowak *et al.* [57], [58] and Popow *et al.* [59], who reported increased β-actin levels in two invasive cell lines, the LS180 human colon adenocarcinoma cell line, and Morris 5123 rat hepatoma cells, respectively. This suggested possible roles for β-actin in tumour metastasis formation.

In 1998, Fages *et al*. [60] examined the β-actin distribution in C6 glioma cells. They showed localised β-actin mRNA at the leading edges in 80% of cells analysed. On the other hand, Panopoulos *et al.* [61] demonstrated that GBM cells can show exceptional motility that can even occur in the absence of polymerised actin. They concluded that even though actin polymers are not totally necessary for glioma cell migration, they do promote the migration of these cells.

By investigating different brain regions of five GBM patients, Com *et al.* [62] identified 24 proteins that were up-regulated in the glioblastoma region, one of which was β-actin. To our knowledge, this was the first demonstration of a direct correlation between β-actin and GBM.

Although data the literature are still controversial, our findings support the idea that β-actin might have a role in GBM cell migration, a finding that should now be considered and carefully examined. As tumour invasion is a disease of dysregulated cell motility, β-actin might play a crucial part in this process.

In conclusion, by using technology based on camelid heavy-chain antibodies we produced a phage-displayed VHH library and after a number of immunoaffinity selections we identified two new potential biomarkers, TRIM28 and β-actin, specific to GBM stem-like cells. The advantage of the approach is the simultaneous acquisition of both antigen and corresponding nanobody that can be used for further development of diagnostic and/or targeting strategies.

## Supporting Information

Figure S1 **Western blot analysis of isolated protein fractions.** The presence of the cytosolic proteins can be detected in all of the isolated protein samples (band at approximately 35 kDa), while the membrane protein-enriched fraction (band at approximately 135 kDa) presents stronger signal in the corresponding samples when compared to the cytosolic fractions from the same biological samples. Samples: GSCm – membrane protein-enriched extract isolated from GBM stem-like cell lines; GSCc – cytosolic/nuclear protein fraction isolated from GBM stem-like cell lines; GBMm – membrane protein-enriched extract isolated from GBM tissues; GBMc – cytosolic/nuclear protein fraction isolated from GBM tissues; NBTm – membrane protein-enriched extract isolated from human brain samples; NBTc – cytosolic/nuclear protein fraction isolated from human brain samples; GBMw – whole protein extract isolated from GBM tissues; NBTw – whole protein fraction isolated from human brain samples.
(TIFF)

Figure S2 **Structural classification of human tripartite motif (TRIM) subfamilies** [45] **.**
(TIFF)
