# Supplementary figures and images for: TRIM28 and β-Actin Identified via Nanobody-Based Reverse Proteomics Approach as Possible Human Glioblastoma Biomarkers
Source: PLoS One. 2014 Nov 24;9(11):e113688. doi: 10.1371/journal.pone.0113688 (PMC4242679; doi:10.1371/journal.pone.0113688)

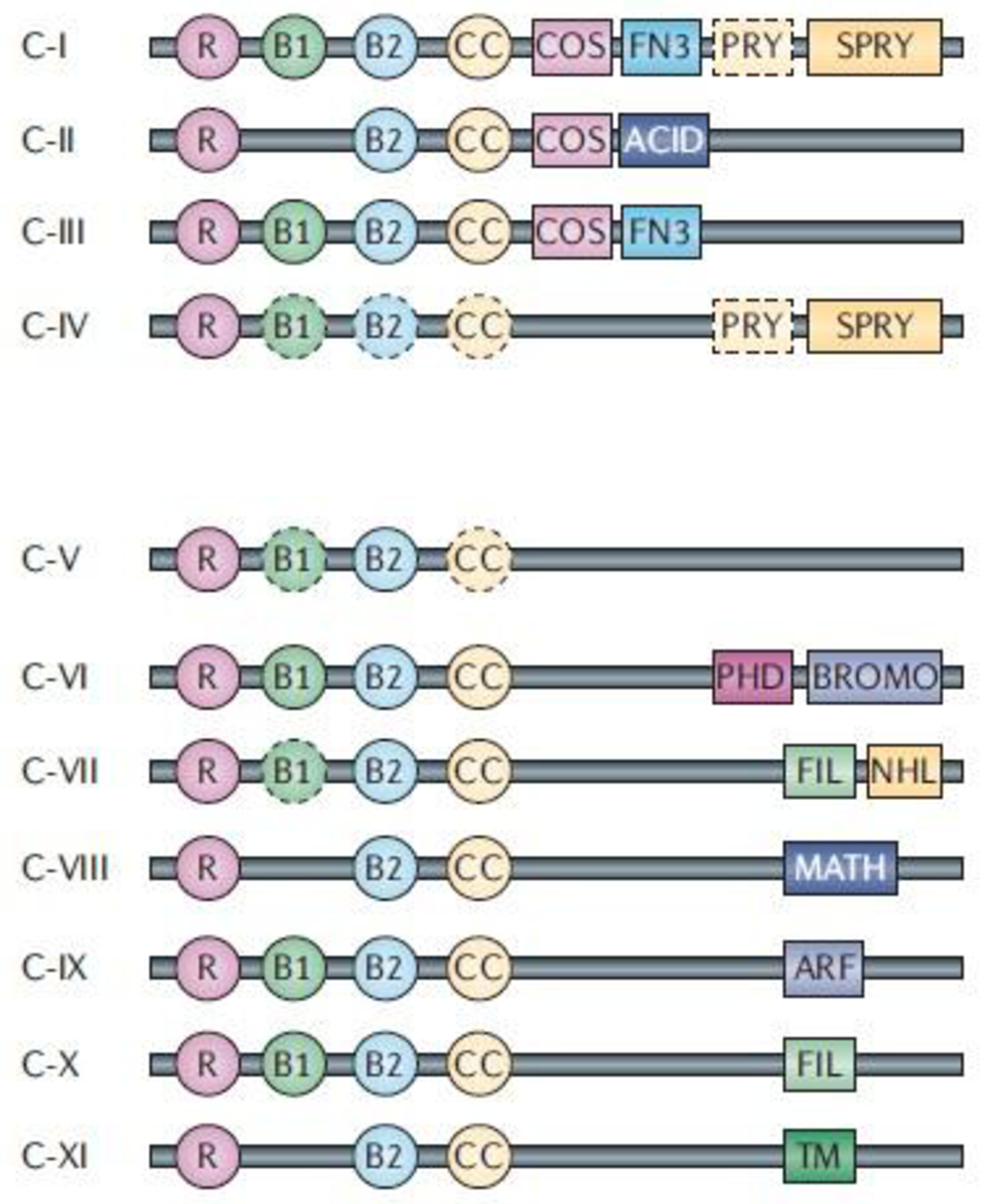

Supplement: Figure S2 — Structural classification of human tripartite motif (TRIM) subfamilies [45] . (TIFF) [file pone.0113688.s002.tiff]
